# Theoretical Analysis of Sequencing Bioinformatics Algorithms and Beyond

**DOI:** 10.1145/3571723

**Published:** 2023-06-22

**Authors:** Paul Medvedev

**Affiliations:** Department of Computer Science and Engineering and the Department of Biochemistry and Molecular Biology and the Director of the Center for Computational Biology and Bioinformatics at Pennsylvania State University, University Park, PA, USA.

When I ask first-year computer science undergraduate students how to quantify the speed of an algorithm, they look at me puzzled and tell me to just run the algorithm and see how long it takes. Such empirical analysis, in fact, is the most direct and natural way to measure algorithm performance. However, it has long been understood to have many shortcomings.^45^ To overcome these shortcomings, computer scientists developed ways to theoretically analyze algorithm performance. The most common technique for this is traditional worst-case analysis. For example, we say merge sort runs in *O* (*n* log *n*) worst-case time, which formally means that there exists a constant *c* such that for any large-enough input of *n* elements, merge sort takes at most *cn* log *n* time. Other more sophisticated techniques, such as parametrized analysis, average-case analysis, or semi-random models, better capture the properties of real data.^44^ Additionally, theoretical analysis can be used to measure not just speed but other aspects of algorithm performance, such as memory usage or accuracy. When undergraduate students take an algorithms course, they finally learn about the theoretical analysis of algorithms and how to use it to capture general patterns of performance that empirical analysis does not.

The most direct impact of such theoretical analysis is in applied algorithms, that is, algorithms implemented and applied to real data (in contrast to complexity theory, where such analysis serves the purpose of understanding the hierarchy of problem, rather algorithm, complexity). The goals of the theoretical analysis of applied algorithms, denoted here as TA3, are twofold:^44^ One goal is to *predict* the empirical performance of an algorithm, either in an absolute sense or relative to others. The second goal is to be a yardstick that drives the design of novel algorithms that perform well in practice. TA3 has achieved its goals with resounding success, being directly responsible for the design and performance prediction of many algorithms used in practice (for example, Dijkstra’s shortest path algorithm). Because of this success, algorithms instructors typically jump into theoretical analysis with only a cursory justification of why it is needed. To put it bluntly, the fact that TA3 achieves the two goals has become a dogma of computer science.

However, fast-paced application domains pose a challenge to TA3. In this article, I use the field of sequencing bioinformatics (SeqBio) as a case study, where I posit that TA3 has failed to achieve its stated goals. SeqBio is an interdisciplinary field that uses algorithms to extract biological meaning from sequencing data. SeqBio has revolutionized the life sciences, with algorithms developed by computer scientists (for example, Bankevich et al.^3^ and Langmead et al.^23^) enabling projects such as the Earth Microbiome Project,^15^ the Vertebrate Genomes Project,^42^ and the Cancer Genome Atlas.^18^ It is also in the process of revolutionized healthcare, enabling projects such as Obama’s Precision Medicine Initiative. However, the vast majority of SeqBio papers either do not perform TA3 (for example, Bankevich^3^) or perform traditional worst-case analysis only to conclude that it is not a good predictor of the algorithm’s performance in practice (for example, Marschall et al.^29^). I will demonstrate two concrete examples of TA3’s failure in SeqBio: the problems of genome assembly and structural variation detection. I will also give one encouraging example of success: *k*-mer data structures and then catalog some of the challenges of applying theoretical analysis in SeqBio, argue why empirical analysis is not enough, and offer a vision for improving the relevance of theoretical analysis to SeqBio. By recognizing the problem, understanding its roots, and providing potential solutions, this work can hopefully be a crucial first step toward making TA3 more relevant in SeqBio and other fast-paced application domains.

Before proceeding, it is useful to make the distinction between direct and indirect influence of TA3 in SeqBio. Without question, TA3 can be credited with the development and analysis of methods which have become part of SeqBio’s toolbox, for example, integer linear programming, clustering algorithms, sketching techniques, and machine learning. For the two problems we will investigate, combinatorial algorithms for optimization problems with theoretical guarantees form the backbone of many tools^3,13,35,46^ for assembly and for structural variant detection.^31^ However, this article is concerned with direct influence, that is, situations where theoretical analysis was applied to problems specific to SeqBio, or at least for which SeqBio was a major motivation.

## Accuracy of Genome Assemblers

SeqBio algorithms work with data generated by various instruments that repeatedly sample short substrings (called *reads*) from a long genome sequence.^28,a^ The locations are chosen semi-uniformly at random but are not output by the instrument; the output only contains the string sequence of each read. Moreover, the reads may contain errors and hence not be exact substrings of the genome. Such a sequencing experiment can generate billions of reads at ever-decreasing costs. There are many applications of sequencing technology, but here I focus on the genome assembly problem, a classical SeqBio problem.

The genome assembly computational problem is to take a sequencing experiment from a single genome and to reconstruct the full DNA sequence of that genome.^47^ There are dozens of widely used assemblers, with hundreds more at the prototype stage. Genome assembly algorithms have enabled genome-wide studies of thousands of species and have a biological impact that is difficult to overstate. The most important aspect of an assembler’s performance is its accuracy, since the resources required to collect a DNA sample usually outweigh the computational resources of an assembler. In this section, we will describe the various attempts to apply theoretical analysis to predict the accuracy of assemblers and design more accurate assemblers.

**Figure 1.**
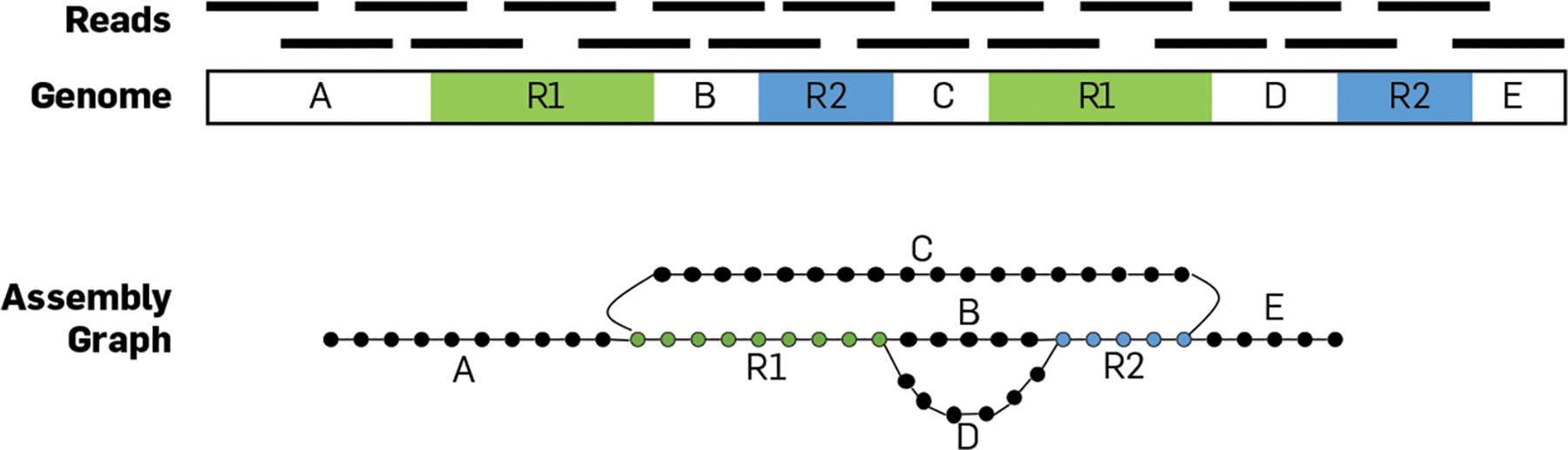
Illustration of a simple assembly algorithm. The figure shows a hypothetical genome composed of several long segments. Two segments are repeated: the green segment R1 appears twice, as does the blue segment R2. The other segments are assumed to be repeat-free, in the sense that all the substrings of length above a certain threshold *k* are unique. A potential set of sampled reads is shown above the genome, lined up according to where they come from in the genome. Their location is not known by the algorithm but is shown here for clarity. A simple assembly algorithm would construct an assembly graph from the reads, shown at the bottom. The nodes are all the *k*-mers (that is, substrings of length *k*) appearing in the reads and there is an edge between a pair of *k*-mers that follow one another in at least one read. The genome is a walk in this graph that covers all the vertices (A, R1, B, R2, C, R1, D, R2, E), however, there is more than one walk with this property (for example, A, R1, D, R2, C, R1, B, R2, E). A simple assembly algorithm would not risk making a mistake and would output only the walks in the graph that it is confident appear as sub-walks of any genome walk. In this case, the output could be the spelling of the seven walks A, B, C, D, E, R1, and R2.

Figure 1 illustrates an example of a simple assembly algorithm. However, several practical factors complicate this simple picture. First, reads can have sequencing errors which introduce erroneous vertices and edges into the assembly graph. Second, some parts of the genome are not covered by reads, introducing gaps in the graph. Third, repetitive sequences are prevalent and make the graph structure more convoluted.^21,33^ These and other factors make it challenging to keep the output of the assembler accurate.

One of the earliest theoretical measures of accuracy is the likelihood of the reads given an assembly. The idea of building an assembler to maximize this likelihood was originally proposed in Myers^32^ and later pursued in Boza et al.,^5^ Howison et al.,^16^ Medvedev et al.,^30^ and Varma et al.^51^ Much of the work centers on finding an appropriate likelihood function, that is, one which models the intricacies of the sequencing process. Unfortunately, these formulations have not directly led to any state-of-the-art assemblers, leaving the design goal of TA3 unfulfilled. The reasons for this are not clear from the literature, but I will discuss later.

I am not aware of any work that attempted to use likelihood to theoretically predict algorithm performance. Some works did explore the idea of using a likelihood score to evaluate the accuracy of an assembly.^12,14,17,24,40,52^ Though these tools have been widely used, they are not designed to give a theoretical accuracy of an assembler but, rather, to be run on a concrete output. As such, they cannot predict in advance how an assembler will empirically perform, leaving the prediction goal of TA3 unfulfilled.

A later approach^7^ is to evaluate an assembler by the conditions under which it can fully reconstruct the original genome sequence. Such conditions could, for example, be the number of reads needed or the highest error that could be tolerated. This accuracy framework did lead to the design of a new assembler called Shannon that has been used in practice,^19^ thus partially fulfilling the design goal. However, these conditions rarely arise in practice (that is, the genome usually has too many repetitive sequences to be reconstructed completely and unambiguously). Therefore, it is not clear if designing algorithms to optimize this would lead to other assemblers that perform well in practice. In terms of the prediction goal, this framework was also applied to theoretically predict the accuracy of some simple assembly strategies;^7^ however, it has not been applied to predict the accuracy of any other assemblers used in practice. The common challenge to all prediction attempts such as this one is that most assembly algorithms rely heavily on ad hoc, hard-to-analyze heuristics.

One more recent approach is to measure what percentage of all the substrings that could possibly be inferred to exist in the genome are output by the assembler.^50^ However, this framework has proven technically challenging to apply to real data, for example, to account for sequencing errors and gaps in coverage. It has not yet led to the design of a new assembler or to the accuracy prediction of assemblers, though work is ongoing.^8^

In summary, theoretical analysis of assembler accuracy cannot be credited with the design of any of the widely used assemblers, nor has it led to any theoretical analysis that can predict the accuracy of an assembler on real data. In practice, assemblers are designed heuristically to perform well on a set of empirically observed metrics,^34^ such as the lengths of the segments that the assembler reports or the recovery of genes whose sequences are conserved across different species.^43^ Additional validation is performed by measuring the agreement with data from an orthogonal sequencing technology, where the sequencing errors have different patterns.^41^ Assembly algorithms are usually developed to perform well on publicly available datasets and often validated by the exact same datasets.

The shortcomings of TA3 have been felt in practice. The Assemblathon 2 competition^6^ performed an empirical evaluation of assemblers and found the ranking of different assemblers according to their relative accuracy depended on the dataset, on the evaluation metrics being used, and on the parameter choices made in the evaluation scripts. These are exactly the shortcomings of empirical evaluation that TA3 is intended to address. Moreover, the assemblers themselves are simply not as good as they could be, or, as one of the reviewers concluded, “on any reasonably challenging genome, and with a reasonable amount of sequencing, assemblers neither perform all that well nor do they perform consistently.”^49^ In the last five years, the practical situation has to some extent improved due to newer sequencing technologies that generate higher quality data. Nevertheless, the experience of the Assemblathon 2 competition is instructive to appreciate the limitations of solely empirical analysis in SeqBio.

## Accuracy of Structural Variation Detection Algorithms

Once a genome is assembled for a species, it forms what is called a reference genome. Follow-up studies then sequence different individuals of the same species but do not perform a de novo genome assembly. Instead, they catalog the variations between the sequenced genome and the reference, under the assumption that the genome is unchanged in places where there is no alternative evidence. Variants that affect large regions of more than 500 nucleotides, called structural variants, are responsible for much genomic diversity and are linked to numerous human diseases, including cancer and myriad neurodevelopmental disorders.^53^ Algorithms for detecting structural variation started to appear around 2008 (see Mahmoud et al.^26^ and Medvedev^31^ for surveys) and a recent assessment identified at least 69 usable tools.^22^ As with genome assembly, the most important aspect of algorithm performance is accuracy.

**Figure 2.**
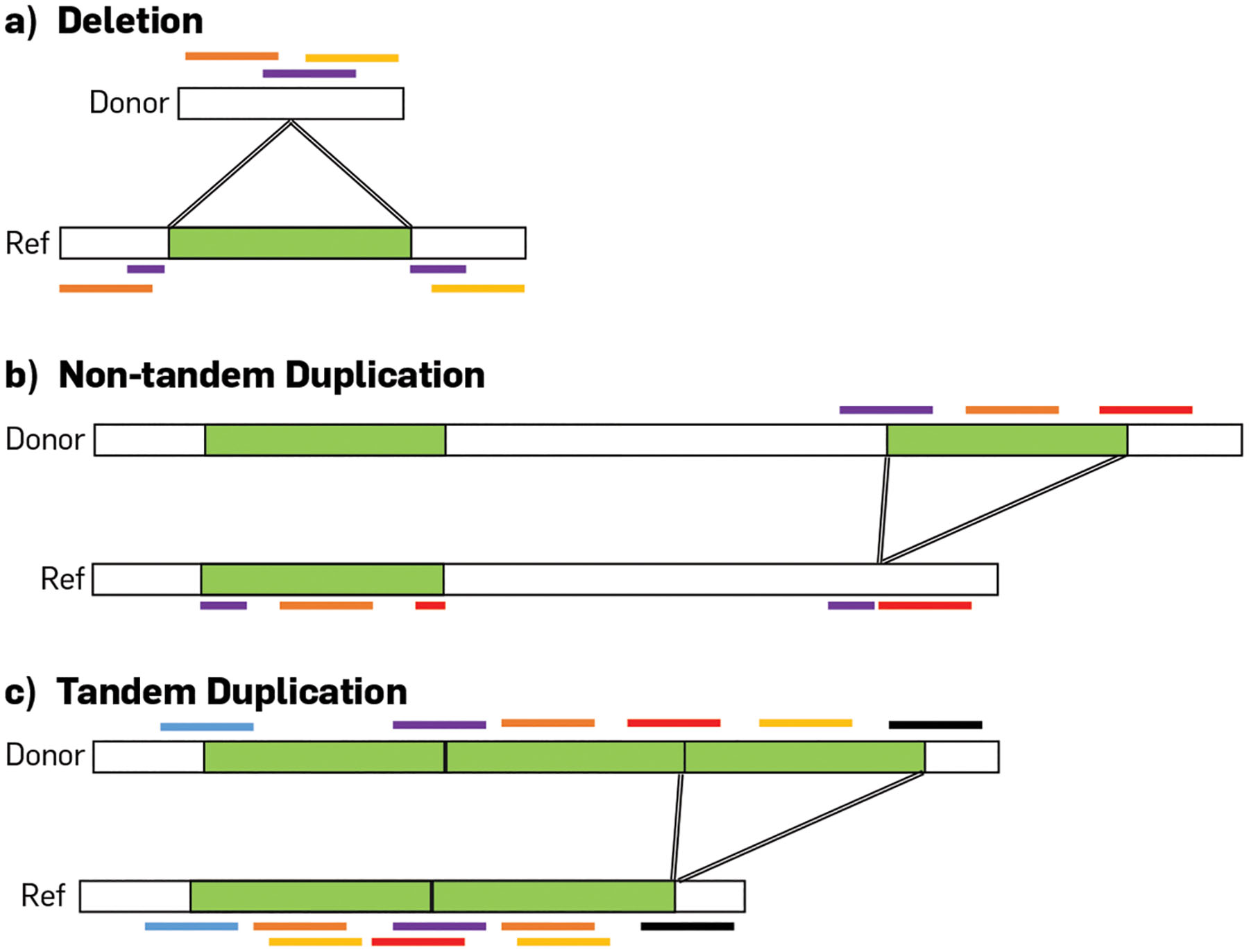
Illustration of structural variation detection. Each panel shows the sequenced donor genome (top rectangle) and the species reference genome (bottom rectangle). It shows the reads above the donor genome according to their origin position; it shows the reads below the reference according to where a string pattern matching algorithm would place them (in more technical terms, an alignment). In panel (a), the donor genome has the green region deleted; in panel (b), the donor has the green region duplicated and inserted far away in the genome; in panel (c), the reference has two copies of the green region while the donor has three. Observe how each event leaves behind a signature that can be detected by an algorithm. In (a), the purple read is split into two partial matches, the position of each indicating the boundary of the deletion. In (b), the purple and red reads each have two partial placements, with one end at the insertion location and the other at the edge of the duplicated sequence. In panel (c), all the reads have normal-looking placements; however, there are more reads mapping to the green region then one would expect if there was no duplication.

Figure 2 illustrates the types of signatures that algorithms can use to detect structural variants. What complicates the algorithm’s task is that the same signature can sometimes be explained by alternate events, the signatures of multiple events can overlap, and repetitive sequences can make it difficult to find the correct location of a read.

Most tools are heuristics with no theoretical analysis of their accuracy. There are some exceptions, when a probabilistic formulation is used to achieve a desired false discovery rate (for example, Marschell^29^). In such cases, TA3 can take some credit for the design of the algorithm. However, accuracy greatly depends on the type of variant (for example, deletions are easier to detect than duplications) and on the location of the variant (for example, repetitive sequences make variants harder to detect). Thus, a useful analysis of accuracy requires a statistical model for the distribution of variant type, location, and relative frequency. But coming up with realistic models is challenging, as our understanding of the biological process that generates structural variants is limited. Therefore, even when accuracy is predicted theoretically, it does not correspond to what is observed in practice because the models are too idealized.^29^ Thus, even in the limited cases where TA3 has been applied, it has not achieved its prediction goals.

As with genome assembly, the algorithms used in practice suffer from many of the limitations that TA3 is intended to address. Algorithms are typically evaluated empirically, using simulated data or an established benchmark. Two recent studies assessing the empirical accuracy of algorithms^9,22^ found the tools suffered from low recall and the ranking of the tools according to accuracy varied greatly across different subtypes of variants. Empirical evaluation is hampered by the same lack of models that hampers theoretical evaluation and Cameron et al.^9^ warned developers against considering “simulation results representative of real-world performance.” In fact, accuracy on simulated data is typically much higher for most tools then on real data.^22^ As with genome assembly, the problems described in Cameron^9^ are the types inherent to empirical-only evaluation: “But with newly published callers [algorithms] invariably reporting favourable performance, it is difficult to discern whether the results of these studies are representative of robust improvements or due to the choice of validation data, the other callers selected for comparison, or over-optimisation to specific benchmarks.”

## Memory Usage of *k*-Mer Data Structures

Sequencing data is often reduced to a collection of *k*-long strings (called *k*-mers) that are stored in a variety of data structures. For example, breaking reads into constituent *k*-mers is part of many assembly algorithms (see Figure 1). The exact data structure depends on the types of queries that need to be supported, the type of associated data maintained, and the source of the *k*-mer set. Examples of queries include simple membership queries (such as, is a *k*-mer present in the data structure?) and group membership queries (Does a given bag of *k*-mers have at least 70% of its *k*-mers present in the data structure?). Examples of associated data include count information (for example, how often does a *k*-mer occur in a set of reads?) or experiment information (Given multiple experiments, which experiments contain the *k*-mer?). Data structures to store *k*-mers have become ubiquitous in SeqBio and form the backbone of hundreds of tools (for surveys, see Chikhi et al.,^10^ and Marchet et al.^27^). The theoretical analysis of their memory is a rare bright light in the theoretical analysis of SeqBio algorithms and I discuss it here to illustrate TA3’s potential for success in SeqBio.

Many of the techniques used to analyze the memory used by *k*-mer data structures have been borrowed from the field of compact data structures.^36^ The analysis of compact data structure memory differs from traditional worst-case analysis in that the higher-order terms are often written without asymptotic notation (for example, 4*n* + *o* (*n*) instead of *O* (*n*)). This helps distinguish algorithms whose memory differs by a constant factor, at the expense of a more technically involved analysis.

Using this type of analysis as a yardstick has led to the design of several *k*-mer data structures that perform well on real data and are included in broadly used software.^1,2,4,11,37,48^ In one example, an analysis led to the design of a compact representation of a popular *k*-mer data structure called the *de Bruijn graph* that uses 4*n* + *o* (*n*) bits, where *n* is the number of edges.^4^ This data structure uses very little memory in practice^39^ and forms the core of the widely used MEGAHIT assembler.^25^ Another successful example of a *k*-mer data structure is the pufferfish index,^2^ which forms part of the popular Salmon^38^ software. In addition to satisfying the design goal, this type of analysis has been used to correctly predict how much memory *k*-mer data structures will use in practice and compare the relative performance of different methods (for example, quantifying the trade-offs between memory and query time of various indices).

Thus, theoretically analyzing memory by retaining the constant for the higher-order terms has satisfied both the design and prediction goals. Such a TA3 success, though rare in SeqBio, demonstrates it is indeed possible for TA3 to have an impact in SeqBio.

## Empirical Analysis Is Not Enough

Despite the limitations of theoretical analysis, SeqBio researchers continuously develop successful tools that have an enormous biological impact. A popular approach that fits both the accelerated development timeline and sidesteps the need for TA3 is to reduce the problem to one from a toolbox of known approaches. A bioinformatician’s toolbox includes black-box solvers for problems like clustering or integer linear programs; it also includes techniques like greedy algorithms or dynamic programming. This toolbox then forms the basis of incrementally designed heuristic algorithms. These are rule-based heuristics where the rules are incrementally improved by looking at where simpler heuristics fails on empirical data. This contrasts with a design driven by a mathematical understanding of the abstract problem with respect to a theoretical yardstick.

Empirical evaluation has its shortcomings but can nevertheless be very beneficial when done right. Benchmark datasets and/or community competitions have been very useful. The Genome in A Bottle consortium for example has released both a benchmark sequencing dataset and a benchmark validation dataset for the problem of structural variant detection. Similarly, competitive assessment contests, such as Assemblathon^2,6^ are designed to test submitted algorithms on strategically designed datasets. Such benchmarks and competitions aim to achieve similar design and prediction goals as TA3. Empirical analysis has in fact been the major driver of algorithm design in all the three examples covered here.

Nevertheless, SeqBio suffers from serious problems that are difficult to resolve with empirical analysis, as discussed earlier. In many cases, benchmarks and competitions have not been developed, or have been developed years later than many of the algorithms (for example, in structural variation). In these cases, there is a proliferation of algorithms that perform well in the empirical validation of the authors but not in an independent evaluation. Note that the intent of the authors is not usually malicious; it is simply that the empirical validation approaches at their disposal are limited due to the lack of benchmarks. Even after the appearance of good benchmarks and competitions, algorithms that do well on these do not necessarily generalize well to other datasets; in fact, the incentives sometimes favor algorithms that over-fit the data. Some assemblers, for example, are known to *perform* better on human or E. coli, which are exactly the benchmarks commonly used for design and evaluation. On the other hand, algorithms that are designed to do well on a good (that is, matching empirical observation) theoretical yardstick have the potential to be more generalizable, and the theoretical yardstick has the potential to predict algorithm performance on different datasets in a way that an empirical benchmark cannot. Thus, TA3 can overcome the problems associated with relying solely on empirical analysis.

## The Challenges of Theoretical Analysis in Sequencing Bioinformatics

Based on the preceding three examples, we can speculate on what factors have made theoretical analysis more challenging for the accuracy of genome assemblers and structural variation detectors than for the memory of *k*-mer data structures. First, computer science is historically more applied to predicting memory rather than accuracy, which is more in the domain of statistics. Second, the two unsuccessful examples are closer to real data than the successful one, that is, they are more subject to the whims of poorly understood biological processes. Finally, it could simply be that compact data structures were a well-developed field before its application to SeqBio and bioinformaticians exploited that.

Moving away from the concrete examples of this article, what is it about an application domain that makes the direct application of TA3 so challenging? I can identify at least five challenges that are characteristic of SeqBio but are general enough to possibly be present in other fast-paced application domains. First, traditional worst-case analysis is in most cases too pessimistic when it comes to real data and fails to separate high-performing algorithms, which take advantage of the structure of real data, from poorly performing ones that do not. This is in fact what many empirically successful algorithms do, as they stem from a deep understanding of the data followed by heuristics to exploit its structure. Such heuristics are difficult to analyze and are unlikely to be invented when the yardstick is traditional worst-case analysis.

Second, because applied researchers require a broad inter-disciplinary skillset, they often lack the technical expertise necessary to apply more sophisticated TA3 techniques. These techniques, sometimes called beyond worst-case analysis, do in fact capture some of the complexities of real data.^44^ A good example of such a technique is smoothed analysis or, more generally, semi-random models, which make the analysis more realistic by assuming there is random noise forced upon any worst-case instance. However, these advanced techniques are rarely taught as part of the core CS curricula, and applied researchers are typically exposed to theory only through introductory courses. This affects the technical complexity of the TA3 that they can perform. For example, it is a rare case that a SeqBio researcher can do a smoothed analysis of an algorithm. Being able to come up with a novel analysis technique is even more rare.

The third reason is that dataset sizes are growing at a rate faster than Moore’s Law.^20^ The usual justification for ignoring constants in traditional worst-case analysis is that a constant factor improvement in time or memory utilization will quickly become obsolete, as computing capacity grows exponentially. But when the size of the data is growing faster than the computing capacity, a constant factor speedup may in fact be relevant for a long time. This helps explain the success of the theoretical analysis of *k*-mer data structures, where the constant is kept.

The fourth reason is that algorithms in a fast-paced application domain are usually developed, analyzed, and applied under significant time pressure. In many cases, the algorithmic problem that a researcher is tasked with solving is only a small part of a more complex project in the application domain (for example, biology). Because the data and its underlying technology is rapidly evolving and changing, time is of the essence. The researcher must work under time pressure to deliver a method that would analyze the data at hand and cannot afford to dedicate months of time to theoretically analyze an algorithm. There are some notable exceptions of SeqBio subareas where the data is stable enough so that the analysis and development of new algorithms has had enough time for complex TA3 techniques to emerge (for example, the edit distance problem), but these cases are rare.

The fifth reason is that there is often an incentive to publish in venues belong to the application domain rather than in CS venues. In SeqBio, for example, a paper will typically have more visibility if published in a biology journal rather than a CS bioinformatics conference. Domain scientists, however, rarely appreciate the difficulties of TA3, even if they lead to empirical breakthroughs. This especially affects early-career practitioners, who are incentivized to maximize visibility.

Because of these challenges, a useful TA3 technique must not only be predictive of empirical performance but also be easy to apply, easy to explain, and easy to understand. Thus, the theoretical analysis of algorithms in fast-paced application domains not only favors but in fact requires simplicity of the analysis technique. A simpler technique will be more trusted by domain scientists (for example, biologists), more broadly understood and applied by practitioners, more easily taught to students, and more likely included in training curricula. The challenge is to have a simple technique which nevertheless accurately captures empirical performance and is an effective yardstick for the development of empirically better algorithms.

## A Vision for the Future

The first step to tackling the challenges of TA3 in SeqBio is to recognize Theoretical Analysis of Applied Algorithms as its own research area, distinct from the design of the algorithms themselves. In SeqBio journals or conferences, it is currently seen as a side note of algorithm development. Even in more theoretical SeqBio venues, the value of a TA3 contribution is not always appreciated. While a breakthrough result will likely be appreciated, a paper describing incremental progress on an algorithm is much more likely to be seen favorably than an article describing substantial progress on an analysis technique. Moreover, there is generally an expectation that a SeqBio paper, even a theoretical one, delivers a novel algorithm. In most cases, this is a valid expectation, but it is not always appropriate for TA3 papers. Such publication challenges limit the formulation of TA3 subproblems and are in general detrimental to progress in the TA3 field.

The case study of SeqBio can offer insights into other fast-paced application domains. The challenges highlighted can serve as a basis for reflection in those domains and it would be interesting to compare the SeqBio experience with others. For example, the TA3 techniques needed to tackle the challenges of structural variation detection may be very different from those needed to tackle the challenges of assembly; however, by aggregating these challenges across multiple domains, we may find shared roadblocks and solutions. Recognizing TA3 as a broad research area will help with the formation of a community of like-minded researchers and all the synergies that go along with it. Unfortunately, the current fragmentation of TA3 research across multiple domains has been a bottleneck to progress.

The first step of a TA3 research program could be to survey the literature for successful applications of TA3 techniques. This article has surveyed the techniques in three sub-areas of SeqBio, but a more thorough investigation is likely to turn up some more successful applications even within SeqBio. It will be useful to also identify successful TA3 techniques from areas of bioinformatics that are not based solely on sequencing data, such as whole-genome analysis and phylogeny reconstruction. The toolbox of successful TA3 techniques can then become the starting point of further research. Moreover, it can be added to the bioinformatics curriculum in computer science.

Viewing TA3 as its own research field would allow researchers to focus on retrospective prediction of algorithm performance; that is, to develop TA3 techniques using algorithms where the empirical performance is already known. For example, when the theoretical computer science community tackled the limits of the competitive ratio analysis technique for the online paging problem, the empirically best algorithm was already known; the challenge was to find the right technique to reach the same conclusion.^44^ Similarly, a distinct TA3 research program would not be afraid to tackle the question of performance prediction for short-read assembly, even though the empirically best methods have already been established.

The goal would be to develop TA3 techniques that are simple yet predictive of real-world performance. Within SeqBio, these techniques could propel it forward and enable it to respond more quickly and accurately to the rapid evolution of sequencing technology. Without investment in TA3 research, SeqBio, and other application domains will continue to be hampered by the limitations of solely empirical analysis of performance.

## Footnotes

a In recent years, the reads have become much longer; however, many assembly algorithms predate this advance.

## References

[1] Almodaresi F, Pandey P, and Patro R Rainbowfish: A succinct colored de Bruijn graph representation. In Proceedings in Informatics Algorithms in Bioinformatics 88, 2017, 18:1–18:15. Schwartz R and Reinert K, eds. Schloss Dagstuhl-Leibniz-Zentrum fuer Informatik.

[2] Almodaresi F, Sarkar H, Srivastava A, and Patro R A space and time-efficient index for the compacted colored de Bruijn graph. Bioinformatics 34, 13 (2018), i169–i177. 29949982 10.1093/bioinformatics/bty292 PMC6022659

[3] Bankevich A SPAdes: A new genome assembly algorithm and its applications to Single-Cell sequencing. J. Computational Biology 19, 5 (2012), 455–477. 10.1089/cmb.2012.0021 PMC3342519 22506599

[4] Bowe A, Onodera T, Sadakane K, and Shibuya T Succinct de Bruijn graphs. WABI 7534 LNCS. Springer, 2012, 225–235.

[5] Boža V, Brejová B, and Vinař T Gaml: Genome assembly by maximum likelihood. Algorithms for Molecular Biology 10, 1 (2015), 1–10. 26042154 10.1186/s13015-015-0052-6 PMC4454275

[6] Bradnam KR Assemblathon 2: Evaluating de novo methods of genome assembly in three vertebrate species. GigaScience 2, 1 (2013). 10.1186/2047-217X-2-10 PMC3844414 23870653

[7] Bresler G, Bresler M, and Tse D Optimal assembly for high throughput shotgun sequencing. BMC Bioinformatics 14, S5 (2013). 10.1186/1471-2105-14-S5-S18 PMC3706340 23902516

[8] Cairo M The hydrostructure: A universal framework for safe and complete algorithms for genome assembly. 2020; arXiv:2011.12635.

[9] Cameron DL, Di Stefano L, and Papenfuss AT Comprehensive evaluation and characterisation of short read general-purpose structural variant calling software. Nat. Commun. 10, 1 (July 2019), 1–11. 31324872 10.1038/s41467-019-11146-4 PMC6642177

[10] Chikhi R, Holub J, and Medvedev P Data structures to represent a set of k-long DNA sequences. ACM Computing Surveys 54, 1 (2021), 1–22. 10.1145/3445967 PMC12965495 41798316

[11] Chikhi R and Rizk G Space-efficient and exact de Bruijn graph representation based on a Bloom filter. WABI 7534 LNCS. Raphael B and Tang J, eds. Springer, 2012,236–248. 10.1186/1748-7188-8-22 PMC3848682 24040893

[12] Clark SC, Egan B, Frazier PI, and Wang Z Ale: A generic assembly likelihood evaluation framework for assessing the accuracy of genome and metagenome assemblies. Bioinformatics 29, 4 (2013), 435–443. 23303509 10.1093/bioinformatics/bts723

[13] Gao S, Sung W-K, and Nagarajan N Opera: Reconstructing optimal genomic scaffolds with high-throughput paired-end sequences. J. Computational Biology 18, 11 (2011), 1681–1691. 10.1089/cmb.2011.0170 PMC3216105 21929371

[14] Ghodsi M De novo likelihood-based measures for comparing genome assemblies. BMC Research Notes 6, 1 (2013), 1–18. 23965294 10.1186/1756-0500-6-334 PMC3765854

[15] Gilbert JA, Jansson JK, and Knight R The earth microbiome project: Successes and aspirations. BMC Biology 12, 1 (2014), 1–4. 25184604 10.1186/s12915-014-0069-1 PMC4141107

[16] Howison M, Zapata F, Edwards EJ, and Dunn CW Bayesian genome assembly and assessment by Markov Chain Monte Carlo sampling. PloS One 9, 6 (2014), e99497. 24968249 10.1371/journal.pone.0099497 PMC4072599

[17] Hunt M, Kikuchi T, Sanders M, Newbold C, Berriman M, and Otto TD Reapr: A universal tool for genome assembly evaluation. Genome Biology 14, 5 (2013), 1–10. 10.1186/gb-2013-14-5-r47 PMC3798757 23710727

[18] Hutter C and Zenklusen J The cancer genome atlas: Creating lasting value beyond its data. Cell 173, 2 (2018), 283–285. 29625045 10.1016/j.cell.2018.03.042

[19] Kannan S, Hui J, Mazooji K, Pachter L, and Tse D Shannon: An information-optimal de novo RNA-Seq assembler. BioRxiv, 2016, 039230.

[20] Katz K, Shutov O, Lapoint R, Kimelman M, Brister J, and O’Sullivan C The sequence read archive: A decade more of explosive growth. Nucleic Acids Research 50, D1 (2022), D387–D390. 34850094 10.1093/nar/gkab1053 PMC8728234

[21] Kingsford C, Schatz M, and Pop M Assembly complexity of prokaryotic genomes using short reads. BMC Bioinformatics 11, 1 (2010), 1–11. 20064276 10.1186/1471-2105-11-21 PMC2821320

[22] Kosugi S, Momozawa Y, Liu X, Terao C, Kubo M, and Kamatani Y Comprehensive evaluation of structural variation detection algorithms for whole genome sequencing. Genome Biol. 20, 1 (June 2019), 1–18. 31159850 10.1186/s13059-019-1720-5 PMC6547561

[23] Langmead B and Salzberg SL Fast gapped-read alignment with bowtie 2. Nature Methods 9, 4 (2012), 357–359. 22388286 10.1038/nmeth.1923 PMC3322381

[24] Li B Evaluation of de novo transcriptome assemblies from RNA-seq data. Genome Biology 15, 12 (2014), 1–21. 10.1186/s13059-014-0553-5 PMC4298084 25608678

[25] Li D Megahit v1.0: A fast and scalable metagenome assembler driven by advanced methodologies and community practices. Methods 102 (2016), 3–11. 27012178 10.1016/j.ymeth.2016.02.020

[26] Mahmoud M, Gobet N, Cruz-Dávalos DI, Mounier N, Dessimoz C, and Sedlazeck FJ Structural variant calling: The long and the short of it. Genome Biol. 20, 1 (Nov. 2019), 1–14. 31747936 10.1186/s13059-019-1828-7 PMC6868818

[27] Marchet C, Data structures based on k-mers for querying large collections of sequencing data sets. Genome Research 31, 1 (2021), 1–12. 33328168 10.1101/gr.260604.119 PMC7849385

[28] Mardis ER DNA sequencing technologies: 2006–2016. Nature Protocols 12, 2 (2017), 213. 28055035 10.1038/nprot.2016.182

[29] Marschall T CLEVER: Cliqueenumerating variant finder. Bioinformatics 28, 22 (2012) 2875–2882. 23060616 10.1093/bioinformatics/bts566

[30] Medvedev P and Brudno M Maximum likelihood genome assembly. J. Computational Biology 16, 8 (2009), 1101–1116. 10.1089/cmb.2009.0047 PMC3154397 19645596

[31] Medvedev P, Stanciu M, and Brudno M Computational methods for discovering structural variation with next-generation sequencing. Nat. Methods 6, 11 (Nov. 2009), S13–20. 19844226 10.1038/nmeth.1374

[32] Myers EW Toward simplifying and accurately formulating fragment assembly. J. Computational Biology 2, 2 (1995), 275–290. 10.1089/cmb.1995.2.275 7497129

[33] Nagarajan N and Pop M Parametric complexity of sequence assembly: theory and applications to next generation sequencing. J. Computational Biology 16, 7 (2009), 897–908. 10.1089/cmb.2009.0005 19580519

[34] Narzisi G and Mishra B Comparing de novo genome assembly: The long and short of it. PloS One 6, 4 (2011), e19175. 21559467 10.1371/journal.pone.0019175 PMC3084767

[35] Narzisi G, Mishra B, and Schatz MC On algorithmic complexity of biomolecular sequence assembly problem. In Proceedings of the 2014 Intern. Conf. Algorithms for Computational Biology. Springer, 183–195.

[36] Navarro G Compact Data Structures: A Practical Approach. Cambridge University Press, 2016.

[37] Pandey P, Bender MA, Johnson R, and Patro R A general-purpose counting filter: Making every bit count. In Proceedings of the 2017 ACM Intern. Conf. Management of Data. ACM, New York, NY, 775–787.

[38] Patro R, Duggal G, Love MI, Irizarry RA, and Kingsford C Salmon provides fast and bias-aware quantification of transcript expression. Nature Methods 14, 4 (2017), 417–419. 28263959 10.1038/nmeth.4197 PMC5600148

[39] Rahman A and Medvedev P Representation of k-mer sets using spectrum-preserving string sets. In Proceedings of 24^th^ Inter. Conf. Computational Molecular Biology 12074 LNCS. Springer, 2020, 152–168. 10.1089/cmb.2020.0431 PMC8066325 33290137

[40] Rahman A and Pachter L CGal: Computing genome assembly likelihoods. Genome Biology 14, 1 (2013), 1–10. 10.1186/gb-2013-14-1-r8 PMC3663106 23360652

[41] Rhie A Chasing perfection: validation and polishing strategies for telomere-to-telomere genome assemblies. bioRxiv, 2021. 10.1038/s41592-022-01440-3 PMC9812399 35361931

[42] Rhie A Towards complete and error-free genome assemblies of all vertebrate species. Nature 592, 7856 (2021), 737–746. 33911273 10.1038/s41586-021-03451-0 PMC8081667

[43] Rhie A, Walenz BP, Koren S, and Phillippy AM Merqury: reference-free quality, completeness, and phasing assessment for genome assemblies. Genome Biology 21, 1 (2020), 1–27. 10.1186/s13059-020-02134-9 PMC7488777 32928274

[44] Roughgarden T Beyond worst-case analysis. Commun. 62, 3 (Mar. 2019), 88–96.

[45] Sedgewick R and Flajolet P An Introduction to the Analysis of Algorithms. Pearson Education India, 2013.

[46] Simpson JT and Durbin R Efficient construction of an assembly string graph using the FM-index. Bioinformatics 26, 12 (2010), i367–i373. 20529929 10.1093/bioinformatics/btq217 PMC2881401

[47] Simpson J and Pop M The theory and practice of genome sequence assembly. Annual Rev. Genomics and Human Genetics 16 (2015), 153–172. 25939056 10.1146/annurev-genom-090314-050032

[48] Sirén J Indexing variation graphs. In Proceedings of the 19^th^ Workshop on Algorithm Engineering and Experiments. SIAM, 2017, 13–27.

[49] Titus Brown C Thoughts on the Assemblathon 2 paper; http://ivory.idyll.org/blog/thoughts-on-Assemblathon-2.html.

[50] Tomescu AI and Medvedev P Safe and complete contig assembly through omnitigs. J. Computational Biology 24, 6 (2017), 590–602. 10.1089/cmb.2016.0141 27749096

[51] Varma A, Ranade A, and Aluru S An improved maximum likelihood formulation for accurate genome assembly. In Proceedings of the 1^st^ IEEE Intern. Conf. Computational Advances in Bio and Medical Sciences. IEEE, 2011, 165–170.

[52] Vezzi F, Narzisi G, and Mishra B Reevaluating assembly evaluations with feature response curves: Gage and Assemblathons. PloS One 7, 12 (2012), e52210. 23284938 10.1371/journal.pone.0052210 PMC3532452

[53] Weischenfeldt J, Symmons O, Spitz F, and Korbel JO Phenotypic impact of genomic structural variation: Insights from and for human disease. Nature Reviews Genetics 14, 2 (2013), 125–138. 10.1038/nrg3373 23329113

